# The Role of Food in Disorders of Gut-Brain Interaction: Introduction to a Rome Foundation Working Group Series

**DOI:** 10.14309/ajg.0000000000001744

**Published:** 2022-03-23

**Authors:** William D. Chey, Jan Tack

**Affiliations:** 1 Division of Gastroenterology and Hepatology, Department of Internal Medicine, University of Michigan, Ann Arbor, Michigan, USA;; 2 Translational Research Center for Gastrointestinal Diseases, University of Leuven, Leuven, Belgium.

Food is central to our social interactions and emotional recollections regarding people, moments, and places. Although food is a source of immense pleasure, patients make clear that food is also at the root of many of their digestive complaints (1,2). In 2013, *The American Journal of Gastroenterology* published a series of reviews by a Rome Foundation Working Group on the emerging role of food in the pathogenesis, illness experience, and treatment of what were then referred to as Functional Gastrointestinal Disorders (FGIDs) (3). At the time, this was quite forward thinking because our understanding of the complex relationship between food, behavior, physiology, and illness was in its infancy. The Rome Foundation understood this and commissioned a group of international key opinion leaders who produced a series of concise, evidence-based reviews, which provided busy clinicians and interested clinical investigators with a one-stop shop on the state of the art regarding this important but under-resourced topic (4–9).

Over the ensuing 9 years, our thinking about FGIDs has expanded and evolved. We have an enhanced understanding of the complexity of the pathophysiology of these conditions. Reflecting this, the Rome Foundation has proposed a change in the label applied to FGIDs, which for many had become a 4-letter word. The new label, Disorders of Gut-Brain Interaction (DGBI), retains a 4-letter abbreviation but one that is intended to be more descriptive and less negatively charged for patients and providers (10).

One of the most important advances in our understanding of DGBI involves the interaction between the gut luminal microenvironment and the host (11). Among the constituents of the luminal microenvironment, food plays a key role in digestive health and disease (4–9). In addition to the food's nutritive role, it possesses osmotic and physical properties and exerts effects on enteroendocrine cells, bile acids, immune cells, and the gut microbiome and metabolome. In aggregate, these effects can influence the gut immune system and enteric nervous system leading to alterations in gut motility and sensation and affecting cognitive and affective functions (4,11–15). Dietary adjustments or elimination diets have gained an increasingly prominent place as a treatment option (12–15), initially mainly for irritable bowel syndrome, but now increasingly for other DGBIs, and this is reflected in the composition of this 2022 dedicated article series.

In the introduction to the *AJG* manuscript series published in 2013, we posited, “food is complex.” These words ring even more true in 2022 (16). It is precisely for this reason that the Rome Foundation recommissioned a working group to summarize the rapidly expanding literature on the role of food in the pathogenesis and treatment of a growing list of DGBI. Like the 2013 reviews, we hope this series of updates not only informs clinicians and investigators on the current evidence but also stimulates questions for future research and most importantly helps clinicians to better educate and improve the health of their patients with DGBI. We also hope that these reviews provide useful source documents which aid in the development of the ROME V criteria for DGBI.

We thank the editors of *AJG* for enabling the dissemination of the work of this Rome Foundation Working Group. We also extend our heartfelt gratitude to the authors for their hard work and dedication to this project. We hope that in the menu of offerings, you find information that suits your fancy. Bon Appetit!

## CONFLICTS OF INTEREST

**Guarantor of the article:** William D. Chey, MD.

**Specific author contributions:** All authors participated in the conception, preparation of the first draft, critical revision of subsequent drafts, and approval of the final article.

**Financial support:** None to report.

**Potential competing interests:** W.D.C. is a consultant for AbbVie, Allakos, Alnylam, Ardelyx, Arena, Bayer, Biomerica, Ironwood, Nestle, QOL Medical, Salix/Valeant, Takeda, Urovant Sciences, and Vibrant; has received grant and/or research study funding from Biomerica, Commonwealth Diagnostics International, QOL Medical, and Salix; has stock options in GI on Demand, Modify Health; serves on the Rome Board of Directors; and is a member of the Board of Trustees of the American College of Gastroenterology and Board of Directors of the International Foundation for Gastrointestinal Disorders. J.T. is a consultant for Adare, Alfa Wassermann, Arena, Bayer, Christian Hansen, Clasado, Danone, Devintec, Falk, Grünenthal, Ironwood, Janssen, Kiowa Kirin, Menarini, Mylan, Neurogastrx, Neutec, Novartis, Nutricia, Ricordati Shionogi, Takeda, Truvion, Tsumura, Zealand, and Zeria pharmaceuticals; has received research support from Biohit, Shire, Sofar, and Takeda; and has served on the speaker bureau for Abbott, Allergan, AstraZeneca, Janssen, Kyowa Kirin, Menarini, Mylan, Novartis, Shire, Takeda, Wellspect, and Zeria and is President of the Rome Foundation.
